# Statistically downscaled climate dataset for East Africa

**DOI:** 10.1038/s41597-019-0038-1

**Published:** 2019-04-15

**Authors:** Solomon H. Gebrechorkos, Stephan Hülsmann, Christian Bernhofer

**Affiliations:** 1 United Nations University Institute for Integrated Management of Material Fluxes and of Resources (UNU-FLORES), 01067 Dresden, Germany; 2 Faculty of Environmental Sciences, Institute of Hydrology and Meteorology, Technische Universität Dresden, 01062 Dresden, Germany

**Keywords:** Projection and prediction, Hydrology

## Abstract

For many regions of the world, current climate change projections are only available at coarser spatial resolution from Global Climate Models (GCMs) that cannot directly be used in impact assessment and adaptation studies at regional and local scale. Impact assessment studies require high-resolution climate data to drive impact assessment models. To overcome this data challenge, we produced a station based climate projection (precipitation and maximum and minimum temperature) for Ethiopia, Kenya, and Tanzania using observed daily data from 211 stations obtained from the National Meteorological Agency of Ethiopia and international databases. Moreover, 26 large-scale climate variables derived from the National Centers for Environmental Prediction reanalysis data (1961–2005) and second generation Canadian Earth System Model (CanESM2, 1961–2100) are used. Statistical Down-Scaling Model (SDSM) is used to produce the required high-resolution climate projection by developing a statistical relationship between the large- and local-scale climate variables. The predictors are analysed more than 16458 times and we provided 20 ensembles for the current (1961–2005) and future (2006–2100, under RCP2.6, RCP4.5, and RCP8.5) climate.

## Abstract (summary)

**Table:** 

Design Type(s)	modeling and simulation objective • time series design • data transformation objective
Measurement Type(s)	climate change
Technology Type(s)	digital curation
Factor Type(s)	geographic location • temporal_interval
Sample Characteristic(s)	Ethiopia • climate system • Kenya • Tanzania

Machine-accessible metadata file describing the reported data (ISA-Tab format)

## Background & Summary

Large-scale or global climate models are currently used to advance the scientific knowledge and understanding variabilities and changes in large-scale climate variables^1^. Information obtained from Global Climate Models (GCMs) supports a better understanding of the climate at a global scale^2,3^. The output from GCMs is too coarse (>100 km) to be used in impact assessment studies, adaptation planning, and decision-making process at local or regional scale^4,5^. In addition to the coarse resolution, biases and uncertainties associated with GCMs increase from global to regional and local scales, which limit the suitability and applicability of GCMs in local-scale impact assessment studies^6–9^. Therefore downscaling is required to increase the spatial resolution and reduce biases^1,10^ before climate projections can be used for impact assessment and adaptation planning. During the last few decades, two types of downscaling techniques have been introduced to reduce biases and improve the spatial resolution of GCMs. These are dynamical and statistical downscaling methods. Dynamical downscaling (e.g., CORDEX-Africa, http://www.cordex.org/domains/region-5-africa/) as a climate modeling process includes local information such as topographic features to produce a high-resolution climate projection, for example 50 km for Africa^11,12^. In addition to the complexity of models and high resource requirements, dynamical models still face biases and sensitivity to the boundary condition of GCMs, which limit their use in local-scale impact assessment and adaptation studies^13–15^. However, compared to dynamical models, statistical downscaling models are fast, simple, effective, and require less computational capacities and expenses.

Statistical models are designed to produce a location based weather series, equivalent to station data, by developing a statistical relationship between local-scale (predictands) and large-scale climate variables (predictors). Considering the overall advantages, statistical downscaling models are widely used in impact assessment studies at local and regional scale in sectors such as water resource and agriculture^10,13,15,16^. Statistical models are classified under three categories based on the statistical approaches used; stochastic weather generator^17^, weather typing^18^, and transfer function^19^.

The Statistical DownScaling Model (SDSM)^19^ is one of the most widely used statistical downscaling models, which is developed based on a transfer function and stochastic weather generator. The performance of SDSM was found to be higher than the conventional weather generators^20,21^. In addition, compared to other statistical downscaling models, SDSM possesses better capabilities in capturing rainfall characteristics and maximum and minimum temperature^19,21^. Therefore, SDSM is used to produce a location-based high-resolution climate projection for regions of East Africa (Ethiopia, Kenya, and Tanzania). Regions of Africa, particularly East Africa, are highly vulnerable to changes in climate and climate extremes and more extreme events such as frequent droughts, floods, and heavy rainstorms are projected in the future^22^. Therefore, considering the observed changes and vulnerability of the region to variability (e.g., seasonal rainfall variability) and changes in climate and climate extremes^22,23^ conducting in-depth impact assessment studies at local and regional scale is required to minimize or mitigate impacts in the future through sustainable adaptation measures. However, this type of information is not readily available and producing station based climate projections using SDSM requires observed data with high quality for model calibration and as input to the scenario generator, which is part of SDSM. It is used to generate, after model calibration and validation, an ensemble of synthetic weather series, using daily predictors supplied by a global climate model^15^.

**Fig. 1:**
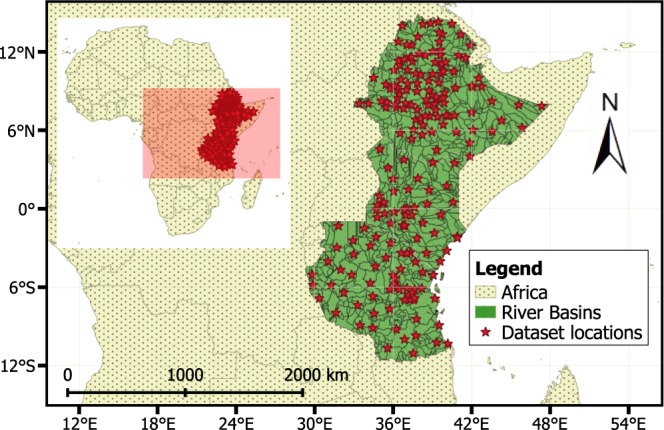
Location map of ground stations and produced datasets (daily precipitation, maximum and minimum temperature) inside river basins (polygons in the map) of Ethiopia, Kenya, and Tanzania.

Availability of observed data from ground-based meteorological stations is, however, limited in East Africa due to issues such as limited temporal and spatial coverage, quality, and accessibility (e.g., data sharing policies). For example, from Tanzania, only five stations with maximum coverage of five-years can be provided by the meteorological agency. Moreover, the Kenyan meteorological agency only provides monthly data, which cannot be used in statistical downscaling. Therefore, a combination of datasets, station data obtained from the National Meteorological Agency (NMA) of Ethiopia and daily data available at the National Centers for Environmental Information (NCEI) are used. For areas with no ground observation (remote and data sparse parts of the region), additional datasets from remote sensing and reanalysis based products with high accuracy (compared with ground station data) and covering a large part of the region are used^24^. Compared to previously developed datasets such as CCAFS (http://www.ccafs-climate.org/data_spatial_downscaling/), which, based on the Delta method, provides a monthly average of 30 years period at different spatial resolution, we provide point information at a daily time scale. Moreover, compared to CCAFS our dataset can be used without restrictions. In this paper, we present statistically downscaled daily precipitation and maximum and minimum temperature for the current climate conditions (1961–2005) and future climate scenarios (2006–2100) under three Representative Concentration Pathways (RCPs; RCP2.6, RCP 4.5, and RCP 8.5). The data can be used for impact assessment and adaptation studies in Ethiopia, Kenya, and Tanzania (Fig. 1).

## Methods

The observed daily precipitation and maximum and minimum temperature data used in this study are the most comprehensive to date in the statistical downscaling process and for this region. Here, we used only stations with higher quality (e.g., concerning missing values) and temporal coverage in order to identify the most dominant predictors and develop the most accurate future climate scenarios.

### Data Acquisition

Observed daily precipitation and maximum and minimum temperature during the period of 1961–2005 is obtained from the National Meteorological Agency (NMA) of Ethiopia and National Centers for Environmental Information (NCEI). For data sparse parts of the region, additional daily precipitation and maximum and minimum temperature (T-max and T-min), based on our earlier study on climate data evaluation for East Africa^24^, are used. For regions with limited availability of station data, climate data products with high spatial and temporal resolution can be used to bridge data gaps^25^. For East Africa, we evaluated different daily climate data sources based on climate models, remote sensing, and reanalysis data and the most accurate data sources are identified for application in climate and hydrological studies. From this study, the Climate Hazards Group InfraRed Precipitation with Station data (CHIRPS)^26^ and Observational-Reanalysis Hybrid^27^ were identified for precipitation and T-max and T-min, respectively (for more information see^24^). In addition, large-scale climate variables (predictors) for the current climate and future scenarios under the RCPs are used, which is available from the Canadian Climate Data and Scenarios (http://climate-scenarios.canada.ca/).

### Downscaling process

SDSM is used to downscale the output from a GCM by developing a statistical relationship between the local (predictands) and large-scale climate variables (predictors) using a multi-linear regressions model and stochastic bias correction techniques^10,15^. Observed daily precipitation and maximum and minimum temperature from 211 stations and 26 predictors (Table 1) from the NCEP (National Centers for Environmental Prediction) reanalysis data and CanESM2 (second generation Canadian Earth System Model) are used. Both the NCEP (1961–2005) and CanESM2 (1961–2100) predictors are available at a spatial resolution of about 2.81°, with nearly uniform longitude and latitude. In a single GCM box, 2–17 ground stations are available for the downscaling process (Fig. 1). Compared to GCMs, the NCEP predictors are commonly used due to their accuracy (e.g., high correlation and Nash–Sutcliffe Efficiency) in representing the current climate^15,28^. Therefore, the NCEP and CanESM2 predictors are used for model calibration and validation and future projection, respectively. The predictors derived from the CanESM2 are available under RCP2.6, RCP4.5, and RCP8.5 for downscaling of future climate projections (2006–2100).

**Table 1:** List of the large-scale climate variables (predictors) used for downscaling.

No.	Long name	Short name
1	Mean sea level pressure	mslp
2	Surface airflow strength	p1_f
3	Surface zonal velocity	p1_u
4	Surface meridional velocity	p1_v
5	Surface vorticity	p1_z
6	Surface Wind Direction	p1th
7	Surface divergence	p1zh
8	500 hPa airflow strength	p5_f
9	500 hPa zonal velocity	p5_u
10	500 hPa meridional velocity	p5_v
11	500 hPa vorticity	p5_z
12	500 hPa geopotential height	p500
13	500 hPa Wind Direction	p5th
14	500 hPa divergence	p5zh
15	850 hPa airflow strength	p8_f
16	850 hPa zonal velocity	p8_u
17	850 hPa meridional velocity	p8_v
18	850 hPa vorticity	p8_z
19	850 hPa geopotential height	p850
20	850 hPa Wind Direction	p8th
21	850 hPa divergence	p8zh
22	precipitation	prcp
23	Specific humidity at 500 hPa	s500
24	Specific humidity at 850 hPa	s850
25	Surface specific humidity	shum
26	Mean temperature at 2 m	temp

**Fig. 2:**
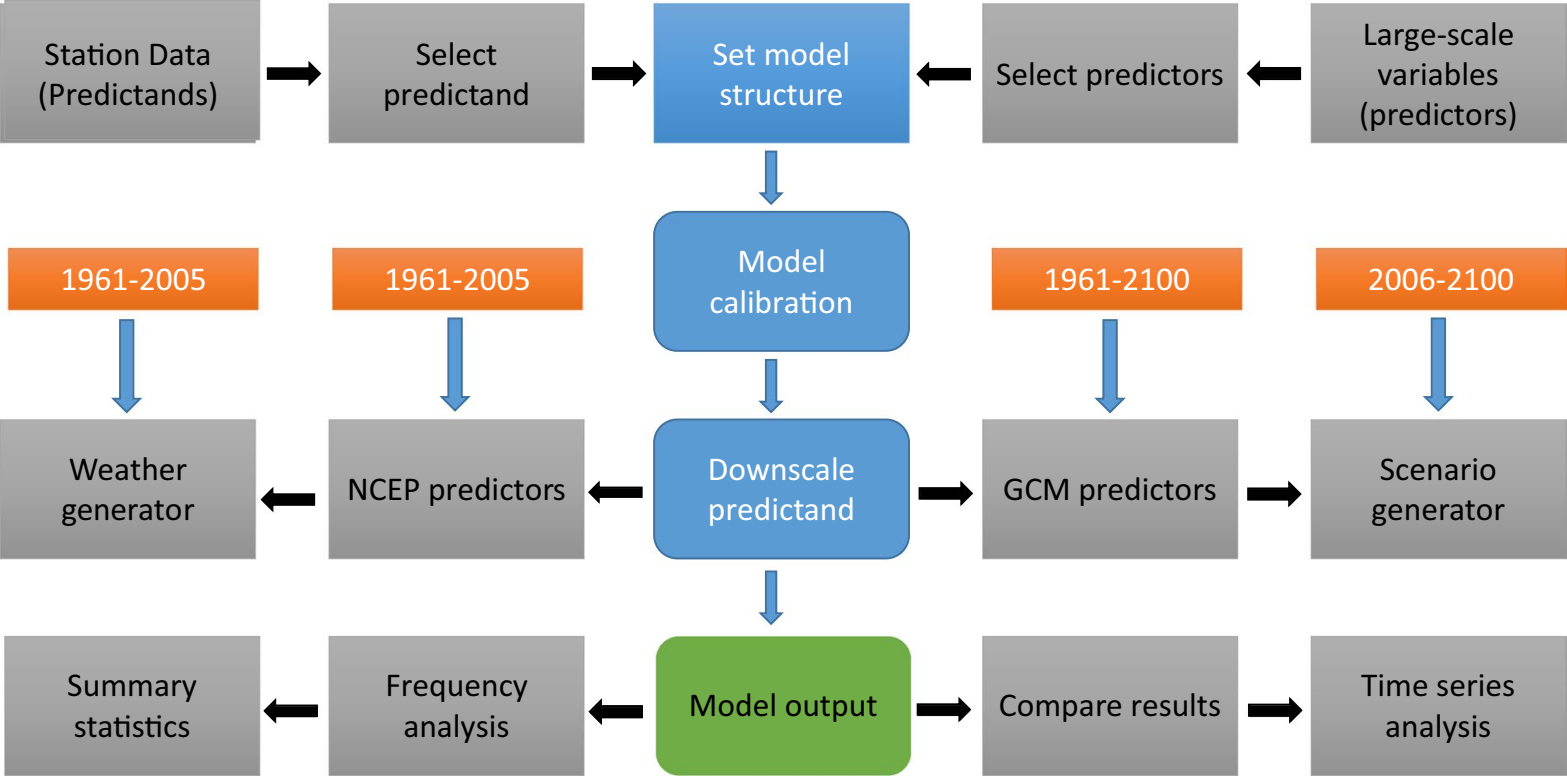
Schematic overview of the Statistical Down Scaling Model (SDSM). Modified from Wilby *et al*.^19^.

After data quality control, predictors are selected for each predictand as shown in Fig. 2. Selection of predictors for a predictand (e.g., maximum temperature) is based on the correlation matrix, partial correlation, and P-value. The selected predictor is further assessed for its accuracy using graphical methods such as a scatterplot. In general, the predictors are analysed more than 5486 (211 stations * 26 predictors) times for a single and 16458 (211*26*3) times for the three predictands (precipitation and maximum and minimum temperature) used in this study.

Using the selected predictors for each predictand, the model is calibrated under unconditional (temperature) and conditional (precipitation) processes on a monthly scale. For stations with a short length of observations, particularly for precipitation, the model is calibrated on seasonal and annual time scales to increase the number of wet days. The calibrated model, using the identified best performing predictors, produces up to 100 ensembles of daily time series and its output is the mean of the ensembles. The model output (ensemble mean) is used to assess the performance of SDSM in reproducing the observed data^10^. The performance is evaluated using a number of statistical parameters (generic and conditional tests) and graphical evaluation methods (e.g., bar plot) included in SDSM. In SDSM, stochastic techniques are included to improve the model performance in reproducing the observed data by artificially inflating the variance of the model output^15^. In addition, optimization techniques such as the ordinary least-square and dual simplex methods are provided in SDSM to control instabilities in regression coefficients^10^. As shown in Fig. 2, the calibrated model is used to generate future scenarios using the CanESM2 predictors available under RCP2.6, RCP4.5, and RCP8.5.

### Data Outputs

The statistically downscaled daily precipitation (Pr), maximum temperature (Tmax) and minimum temperature (Tmin) for the current climate (1961–2005) and future scenarios under the RCPs (RCP2.6, RCP4.5, and RCP8.5) are given in zipped boxes (Box_1 to Box_211). For instance in Box_1, there are 15 text files (.OUT and.PAR) for the three variables as follows;

1. Daily precipitation (Pr)

- Pr.PAR, list of selected predictors for daily precipitation at location one.
- Pr-syn. OUT, model output of daily precipitation for the current period (1961–2005) generated using the weather generator.
- Pr-rcp26. OUT, projected daily precipitation under RCP2.6 (2006–2100).
- Pr-rcp45. OUT, projected daily precipitation under RCP4.5 (2006–2100).
- Pr-rcp85. OUT, projected daily precipitation under RCP8.5 (2006–2100).

2. Daily maximum temperature (Tmax)

- Tmax.PAR, list of selected predictors for daily maximum temperature at location one.
- Tmax-syn. OUT, model output of daily maximum temperature for the current period (1961–2005) generated using the weather generator.
- Tmax-rcp26. OUT, projected daily maximum temperature under RCP2.6 (2006–2100).
- Tmax-rcp45. OUT, projected daily maximum temperature under RCP4.5 (2006–2100).
- Tmax-rcp85. OUT, projected daily maximum temperature under RCP8.5 (2006–2100).

3. Daily minimum Temperature (Tmin)

- Tmin.PAR, list of selected predictors for daily minimum temperature at location one.
- Tmin-syn. OUT, model output of daily minimum temperature for the current period (1961–2005) generated using the weather generator.
- Tmin-rcp26. OUT, projected daily minimum temperature under RCP2.6 (2006–2100).
- Tmin-rcp45. OUT, projected daily minimum temperature under RCP4.5 (2006–2100).
- Tmin-rcp85. OUT, projected daily minimum temperature under RCP8.5 (2006–2100).

In each file, for example, precipitation (Pr-syn. OUT) at Box_1, the model output contains 20 ensembles for the current period. The 20 ensembles produced for each predictand show the uncertainty in the projection and this depends on the selected predictors and predictand and length and quality of observed data. The parameter files (.PAR) only provide the short names of the predictors as shown in Table 1. The inclusion of the predictors selected for each station in this dataset enables researchers to identify the large-scale climate variable linked with the local climate. As East Africa is one of the most topographically complex parts of Africa, the predictors vary considerably from location to location. In addition to the data Zip file, location information (latitude (lat) and longitude (lon)) is given as an excel file (Box_location.csv) for each box.

## Data Records

For Ethiopia, Kenya, and Tanzania, the daily precipitation and maximum and minimum temperature dataset for the current (1961–2005) and future periods (2006–2100, under the RCPs) are available as a zipped file for download^29^. The zipped file contains 15 files for precipitation and maximum and minimum temperature as explained in the above section (data output). In order to make the data easier for reuse, the data is provided in a text format that can be easily read by different programming languages such as R and Python.

## Technical Validation

Evaluation of the model output for both precipitation and maximum and minimum temperature is carried out using the observed data for each station. In SDSM, multiple model evaluation methods (statistical and graphical) methods are included to assess the performance of the calibrated model in reproducing the observed data. As explained in the above section, the performance of the model depends on the selected predictors for the predictand at a given location. Even though a predictor shows a good correlation and low P-value (<0.05) during the screening process, this predictor might not really be the best in reproducing the observed data, which might be due to the presence of outliers. Therefore, a predictor has to be screened first using the correlation matrix, P-value, and scatterplots and the final output is evaluated using the model statistical (e.g., mean, variance and standard deviation) and graphical (bar and line plots) methods. In addition to the statistical parameters available in SDSM, additional methods such as the coefficient of determination (R^2^), Root Mean Square Error (RMSE), and Percent of bias (Pbias) are used^30^ to identify the most accurate predictors. Both R^2^ (Eq. 1) and RMSE (Eq. 2) are indicators of goodness of fit, while Pbias (Eq. 3) shows the tendency of the observed data to be over- or underestimated by the model.

1 $${R}^{2}=\frac{{\sum }_{i=1}^{N}({X}_{i}\,-\,\bar{X})({Y}_{i}\,-\,\bar{Y})}{\sqrt{{\sum }_{i=1}^{N}{({X}_{i}-\bar{X})}^{2}\,{\sum }_{i=1}^{N}{({Y}_{i}-\bar{Y})}^{2}}}$$

2 $$RMSE=\sqrt{\frac{{\sum }_{i=1}^{N}{({Y}_{i}-{X}_{i})}^{2}}{N}}$$

3 $$Pbias=\frac{{\sum }_{i=1}^{N}({Y}_{i}\,-\,{X}_{i})}{\sqrt{{\sum }_{i=1}^{N}{X}_{i}}}\times 100$$

where Xi and $\bar{X}$ and Yi and $\bar{Y}$ are the observed and model monthly and average data, respectively, of the ith event in N number of events.

**Fig. 3:**
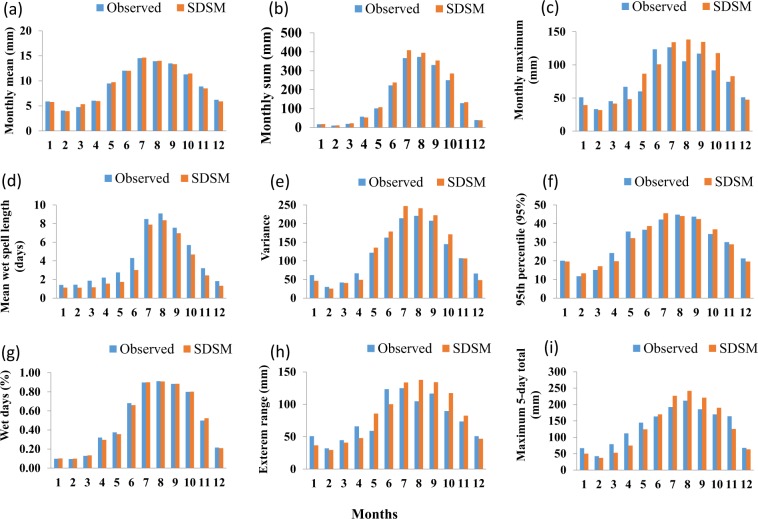
An example of the performance of SDSM compared to the observed monthly precipitation characteristics. (**a**) Mean. (**b**) Sum. (**c**) Monthly maximum. (**d**) Mean wet spell length. (**e**) Variance. (**f**) 95^th^ Percentile. (**g**) Percentage of wet days. (**h**) Extreme range. (**i**) Maximum 5-day total precipitation.

The overall evaluation methods enabled us to accurately identify the best fit predictor for the 211 stations used in this study. An explanatory example for one station in Ethiopia (Nekemt; latitude = 9.08°N, longitude = 36.46°E) is provided in Fig. 3. Figure 3 shows the performance of SDSM in generating some of the station based precipitation characteristics such as the average monthly mean, sum, maximum, wet spell length, variance, 95^th^ percentile, percentage of wet days, extreme range, and maximum 5-day precipitation. For precipitation at station Nekemt, the selected predictors are;

- Mean sea level pressure (mslp),
- Surface divergence (p1zh),
- 850 hPa vorticity (p8_z), and
- Specific humidity at 850 hPa (s850). This shows that the day to day variabilities in mslp, p1zh, p8_z, and s850 are useful predictors for precipitation occurrence at station Nakemet compared to the predictors provided in Table 1.

The results (Fig. 3 and Table 2) shows the accuracy of the model in reproducing the observed precipitation characteristics and shows a high R^2^ and lower biases and errors. Here, the ensembles mean is used to compare with the observed data. As shown in Table 2, the model shows high values of R2 (>0.96) for the selected precipitation characteristics. Modeling precipitation is one of the most challenging climate variables due to the low predictability of by regional climate forcing^15^ and in a topographically complex region.

**Table 2:** An example of model performance for monthly average precipitation and temperature values at station Nekemet during 1961–2005.

Variable	Values	R^2^	RMSE	Pbias
Precipitation	Mean	0.997	0.25	0.1
	Maximum	0.90	18.15	6.1
	Variance	0.99	17.66	4.7
	Sum	0.998	18.99	7.8
	Percentage of wet	0.999	0.012	−0.7
	Wet spell	0.99	0.76	−17
	95^th^ Percentile	0.96	2.36	−0.5
Maximum temperature	Mean	0.99	0.03	0.01
	Maximum	0.82	2.26	1.5
	95^th^ Percentile	0.95	0.88	2.6
Minimum temperature	Mean	0.99	0.1	−0.5
	Maximum	0.67	1.66	5.7
	95^th^ Percentile	0.88	0.69	3.5

**Fig. 4:**
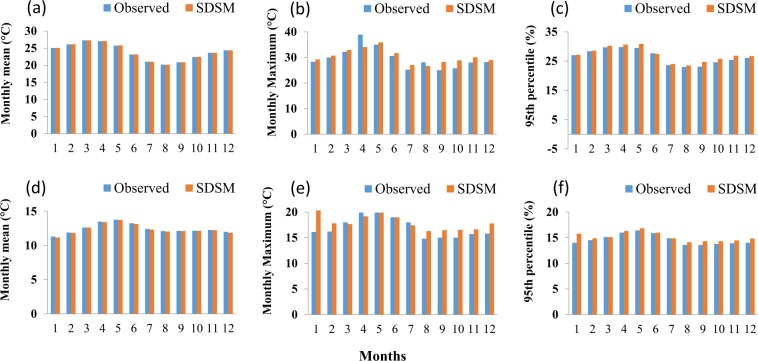
Performance of SDSM compared to the observed monthly temperature for station Nekemet. (**a**) Mean of maximum temperature. (**b**) Maximum of maximum temperature. (**c**) 95^th^ Percentile of maximum temperature. (**d**) Mean of minimum temperature. (**e**) Maximum of minimum temperature. (**f**) 95^th^ Percentile of minimum temperature.

In addition, the model shows high accuracy for maximum and minimum temperature (Fig. 4 and Table 2). Compared to the mean (R^2^ > 0.99), the maximum of maximum and minimum temperature are overestimated by 1.5% and 5.7%, respectively (Table 2). For maximum and minimum temperature the selected predictors are mean sea level pressure (mslp), Surface divergence (p1zh), and 850 hPa vorticity (P8_z) and Surface meridional velocity (p1_v), 500 hPa geopotential height (p500), Specific humidity at 850 hPa (s850), and Surface specific humidity (shum), respectively. In general, the same approach is used to assess the performance of SDSM and to identify the best performing predictors for all the stations used in this study. For the 211 stations, after quality control, the predictors are evaluated more than 16458 times.

Overall, considering the complexity of the variable, particularly modelling of precipitation, the presence of data gaps, and topography of the region, the results are promising and can be used to drive impact assessment and adaptation studies in this region. In addition, SDSM was also identified as an accurate model in infilling missing values in data-sparse regions such as in Africa and the Middle East^25^. Using the new version of SDSM (SDSM 5.2), the data can be also used to assess the vulnerability of location-based adaptation measures and develop climate change scenarios without the dependency of GCMs^31^.

## ISA-Tab metadata file

Download metadata file.

## Data Availability

SDSM version 4.2, freely available (https://sdsm.org.uk/software.html), is used to statically downscale the projection from the second generation Canadian Earth System Model (CanESM2). The predictors derived from CanESM2 and the NCEP reanalysis data^32^ are exported into SDSM directory for model calibration and projection. The CanESM2 is one of the GCMs used in the Coupled Model Inter-comparison Project Phase 5 (CMIP5). A free code written in R (mean-R.txt) is provided to compute the ensembles mean for a single predictand.
